# Perceptions on Healthy Eating Impact the Home Food Environment: A Qualitative Exploration of Perceptions of Indigenous Food Gatekeepers in Urban Fiji

**DOI:** 10.3390/nu15183875

**Published:** 2023-09-06

**Authors:** Shazna M. Buksh, Phillipa Hay, John B. F. de Wit

**Affiliations:** 1 School of Law and Social Sciences, The University of the South Pacific, Suva 1168, Fiji; s.m.buksh@uu.nl; 2 Translational Health Research Institute, Western Sydney University, Penrith, NSW 2751, Australia; p.hay@westernsydney.edu.au; 3 Department of Interdisciplinary Social Science, Faculty of Social and Behavioural Science, Utrecht University, 3584 CS Utrecht, The Netherlands

**Keywords:** home food environment, nutrient transitions, healthy eating, food choices, obesity, health promotion, locally grown foods, food security

## Abstract

The home food environment (HFE) can have important direct and indirect impacts on dietary practices. Nutrient transitions in the HFE of Pacific Island countries (PICs) are key contributors of the high rates of adult and childhood overweight and obesity in the region. Pacifica mothers are important sociocultural agents who play critical roles in their HFE through setting eating-appropriateness standards and mitigating the impacts of food availability and accessibility on the HFE. This study used an interpretative phenomenological approach to explore how urban indigenous Fijian mothers perceive healthy eating and how these perceptions impacted the food decisions they made for their families. Mothers in this study held complex, multifaceted perceptions on healthy eating and these perceptions had both positive and negative impacts on the family food choices they made, the strategies they adopted for healthy eating and their perceived motivators for healthy eating. The findings of this study underscore the need for a deeper understanding and analysis of uptake of public health messaging related to healthy and unhealthy eating and the importance of targeted promotion of healthful nutrition in this community. Promoting consumption of traditional and locally grown foods can enhance nutrition and food security and combat nutrition transition in the region.

## 1. Introduction

The food environment, and in particular the home food environment (HFE), can have important direct and indirect impacts on dietary practices. The HFE comprises overlapping interconnected political and economic environments (e.g., government and business policies, socioeconomic status, family food security), sociocultural environments (e.g., cultural norms, beliefs and practices around eating, nutrition education, exposure to food marketing and advertising) and built and natural environments (e.g., food production, availability and accessibility, access to home and community gardens, access to electronic and smart devices, kitchen appliances and cooking equipment) that impact food intake [1]. This makes the HFE from different cultures and regions unique and while much research has been conducted on the HFE in western and industrialized countries [2,3,4,5,6], there is very poor understanding of the HFE in Pacific Island countries (PICs).

The HFE in PICs, including Fiji, is undergoing nutrient transitions and these are likely a major contributor to the high rates of adult and childhood overweight and obesity in the region [2,3,4,5,6,7,8,9,10,11]. For instance, prior to European contact and colonization, traditional Pacifica diets consisted mainly of starchy root crops, indigenous fruits, green leafy vegetables, coconut, freshwater proteins and seafood [12]. This diet has been replaced by diets high in processed foods which are high in sugar, salt and unhealthy fats [13].

Brug et al. [14] in an extensive review which pooled data from a series of systematic reviews and original studies, argued that sociocultural environmental factors which define what is socially acceptable, desirable and appropriate to eat may be more important in healthy eating than environmental factors such as accessibility and availability (p. 312). They stressed that, in particular, the impact of parenting practices appear pertinent to navigating unhealthy obesogenic environments: Parents are important sociocultural agents who play pivotal roles in encouraging healthy eating through appropriate modelling behavior and parenting practices and styles [14] (p. 301). To illustrate this, they draw upon evidence of how restrictive practices relating to consumption of sugar sweetened beverages has less impact on children who perceive their parents as “less strict” or “involved” [14] (p. 311). More recent studies also support their claim on the importance of parenting styles and practices on healthy and unhealthy eating behavior which have both short term and long-term impacts on weight problems in children, e.g., [15,16,17,18].

Within Pacific literature we find support for both, that is, (1) the role of parents as important sociocultural agents who set eating-appropriateness standards, and (2) how as sociocultural agents, parents mediate the impact of political, economic and physical environments on eating. Studies have consistently reported that Pacifica parents, especially mothers, influence eating patterns and behavior at home by selecting what is appropriate to eat, how much individual family members eat, and when is eating appropriate, e.g., [9,19,20,21,22,23,24]. For instance, Pacifica mothers not only play key roles in selecting food groups [19], but also bear the primary responsibility for shopping for foods and preparing meals for their households [22]. In a large-scale study with 17,150 children, over two thirds of children of Pacifica-descent reported that their mothers played important roles in supporting healthy eating [24]. In comparison, only half of the children of European descent in the study reported this for their mothers, and only half of the children in the study reported this for their fathers [24]. Furthermore, the role of urban indigenous Fijian (iTaukei) mothers as important sociocultural agents who mediate the impact of political, economic and physical environments on their HFE was recorded in a study where mothers reported how they navigated changes in food accessibility and availability during the COVID-19 pandemic [19]. Their reports detailed both positive and negative changes in diet, eating and food purchasing behavior in response to loss of income and government enforced lockdowns during the pandemic [19]. Therefore, Pacifica mothers are important sociocultural agents who play critical roles in the HFE and their perspectives and perceptions of healthy eating can provide important insights into the HFE.

Whilst mothers’ food decisions are influenced by interrelated and overlapping HFE factors [25,26,27,28,29], studies indicate that mothers who value healthy eating and/or aspire to eat healthily, positively impact healthy eating in their families [26,30,31,32]. Studies also suggest that food classifications as “healthy” or “unhealthy” are frequently used for making everyday food decisions by simplifying the process of choosing foods and the management of healthy and unhealthy eating [33,34]. Data from largely Western and industrialized samples reveal several overarching and recurring themes on perceptions of healthy eating: healthy eating has been associated with (1) food type (e.g., fiber-rich foods such as fruits and vegetables, lean meat, foods low in salt, sugar and fats), (2) food quality and preparation (e.g., fresh, unprocessed and homemade foods), (3) concepts around balance, dietary diversity and moderation, and (4) the perceived impact of foods (e.g., on physical and emotional wellbeing) [33,34,35,36,37,38,39,40,41].

However, little is known about how Pacifica mothers perceive healthy eating and how this impacts their food decisions. This is especially important due to the crucial role that Pacifica mothers play in the HFE: if Pacifica mothers, who are a main agent of food selection, preparation, volume determination and major influencers of healthy eating [9,19,20,21,22,23,24] have poor dietary practices or poor understanding of a healthy diet, as suggested from the high rates of obesity and overweight amongst women of reproductive age in the region [8], then the diet and eating practices of the entire family can be poor, making family members prone to obesity. The present paper further contributes to our understanding of the HFE in the Pacific by exploring how urban iTaukei mothers perceive healthy eating and how these perceptions may impact the food decisions they make for their families. We expect that greater insight into how iTaukei mothers conceptualize healthy eating will enable targeted promotion of healthful nutrition in this community through capitalizing on healthy eating attitudes and behavior, and addressing information gaps in knowledge and unhealthy food choices and behavior.

## 2. Materials and Methods

### 2.1. Research Design Overview

Interpretative phenomenological analysis (IPA) was used as a framework for data collection and analysis as it allows for both a detailed exploration of personal perceptions and experiences of iTaukei mothers on healthy eating and an examination of these descriptions and how they impact typical family meals. Due to this two-stage interpretation process, which combines empathetic hermeneutics and questioning hermeneutics [41] (p. 53), IPA has been especially useful in public health research and psychology, e.g., [42,43,44].

### 2.2. Researcher Description

The principal researcher (SMB) is an Indo-Fijian (Fijian of Indian descent) mother residing in the Greater Suva Urban Area (GSUA). She has previous research experience with the urban iTaukei community where she explored (1) the negative and positive changes in diet, eating and food purchasing experiences in this community during COVID-19 safety protocols enforced by the Fijian government [19], and (2) how sociocultural factors impact unhealthy eating, overeating, and nutrition transitions within this community [20]. She also has previous experience with using IPA for exploring perceptions and experiences of stigma and discrimination within iTaukei and Indo-Fijians living with HIV/AIDS in Fiji [45].

### 2.3. Study Site and Population

Fiji is a small PIC with a population of approximately 900,000 [46]. This study was undertaken in the largest urban center in Fiji, GSUA, which is home to approximately a third of Fiji’s population and 57% of Fiji’s urban population [47]. This region has high literacy rates (97%) and at least 80% of women in this region are in paid employment [46]. Fijian urban populations, such as the GSUA not only have easier access to cheap ultra-processed foods and fast foods but also have higher consumption of these foods compared with rural regions in Fiji [48].

### 2.4. Recruitment Process

Purposive sampling was used to recruit mothers who played key roles in food decisions of their families and could provide detailed information on family meals and share their perceptions on healthy eating. Community leaders assisted in recruitment of participants who met the inclusion criteria, resulting in a small and generally homogenous sample, appropriate for IPA. Theoretical saturation was used for determining sample size and interviews were stopped when no new information relating to perceptions of healthy eating was identified after five consecutive interviews.

### 2.5. Participants

Eligible participants were iTaukei mothers involved in family meal planning, shopping and preparation, who possessed sufficient English language skills, and were capable of giving consent. The sample consisted of twelve iTaukei mothers aged 28–49 years (*M* = 38.41, *SD* = 7.55) from the GSUA. All mothers self-identified as Christians and had at least primary school education. Their family sizes ranged from three to ten people. Five of the women lived in extended family settings whilst seven lived with their nuclear families only. Elderly members of extended families reportedly suffered from one or more chronic illnesses such as diabetes and/or high blood pressure and one of the participants was a cancer survivor. Four of the mothers were stay-at-home mothers and the remaining were either working fulltime (*n* = 6) or studying fulltime (*n* = 2). All participants gave homemade school lunches to their children, however mothers and their partners who worked or studied fulltime, normally bought their lunch. Eating out was more common for these families than families with stay-at-home mothers. For the latter group, eating out was reserved for family outings and celebrations.

### 2.6. Data Collection

A semi-structured interview guide with two main sections was used for the in-depth interviews. The first section consisted of questions exploring typical family meals, which was followed by a second section on perceptions of healthy eating. This structure of the interview guide permitted both exploration of the perceptions on healthy eating as well as an examination of how these perceptions might impact family food decisions and, therefore, the HFE. It also ensured that participant descriptions of typical meals were not influenced by considerations of definitions of healthy eating. To ensure a detailed exploration of participant’s experiences and perceptions, questions were open-ended and flexibility was exercised in the phrasing and order of questions within each section.

The interviews were conducted via telephone or Zoom and were recorded with the consent of participants using the audio function in Zoom. All interviews were conducted in English and lasted approximately 40 min. Participants received a reimbursement of FJD 20 through online money transfer at the end of the interview. The interviews were transcribed verbatim within three days of the interview.

### 2.7. Data Analysis

**Figure 1:**
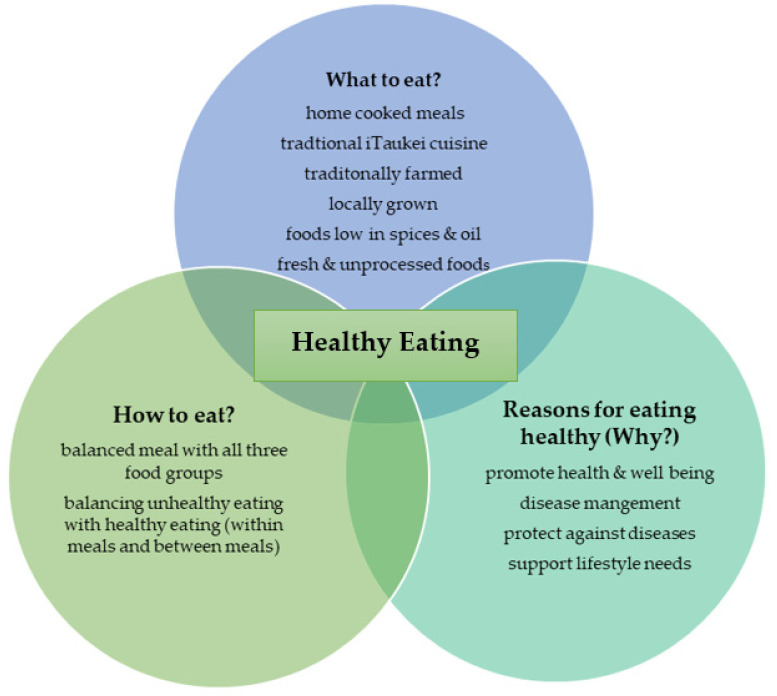
Urban indigenous Fijian mother’s perspectives on healthy eating.

The transcripts were read multiple times and annotated, noting especially the language that was used by the participants. These initial notes were used to identify themes in a transcript and clusters of themes were developed around superordinate themes. For each of the interviews, themes, superordinate themes and directories of participant quotes that supported the themes were identified. Once this process was completed for the eight transcripts, the patterning of themes and superordinate themes was explored across transcripts to identify divergence and convergence, and Figure 1 was constructed. Participant quotes were selected to illustrate the themes. To enhance credibility of the findings, preliminary results were presented and discussed with Authors 2 and 3 and academic colleagues.

### 2.8. Ethical Approval

Ethical approval for the study was granted by the Research and Innovation Office of The University of the South Pacific. To protect participant identities, they were assigned numbers denoting the order in which the interviews took place, prefixed by the letter “P”.

## 3. Results

After exploring their typical meals, mothers were asked to share their perceptions of “healthy eating”. In-depth exploration of mothers’ accounts revealed that they held complex, multifaceted views of healthy eating, centered around three superordinate themes:

1. What foods are healthy (and unhealthy to eat)?
2. How to eat healthily?
3. Healthy eating as means to an end, i.e., to promote health, wellbeing and meet lifestyle requirements.

Figure 1 summarizes the perspectives on healthy eating shared by the participants of this study.

### 3.1. What to Eat: An Emphasis on Traditional Cuisine and Farming Methods

A dominant view shared by the participants was that home cooked traditional iTaukei cuisine, such as boiled roots crops, stews and soups, was healthy, as it uses (1) locally grown food that are perceived as “fresh”, “healthier” and “organic” and (2) cooking methods that do not require oil and spices. In contrast, cuisine from other cultures, including fast food, was viewed as unhealthy because they were “dry foods”, cooked in oil or with a lot of spices.

P5: And I always cook their lunch, at home. We don’t eat outside stuffs like fish and chips. It’s very oily and not good for their [family’s] health. At home we have Fijian [iTaukei] food and I make soups and stews. 

P7: Outside food is fried and has too much oil and spices. At home you can make simple iTaukei food but it is healthy for your family. Mothers should cook food at home for their family.

Some participants (*n* = 3) stressed that, to them, healthy eating went beyond incorporating more traditional cuisine and locally grown foods into their diets. These participants suggested that it is equally important to consider traditional farming and fishing methods. To these women, eating traditional cuisine and using traditional farming methods was also an important aspect of preserving and transmitting their culture and traditions. 

P1: It should be healthy living and eating …, is looks at your food production …, cooking the food, how you make this food and then how you serve this food []. Because indigenous cultures are not just centered around indigenous traditions of knowing, but also indigenous traditions of being, and that includes eating charcoal, roasted breadfruits and that includes eating indigenous organically grown food like bele [leafy greens] and dalo and cassava, and and yams [root crops]. [] We also partake in in a shared legacy if you like, of our forefathers in terms of when we eat organically grown dalo or organically grown cassava and breadfruit.

Therefore, participants characterized large-scale commercial farm produce where many pesticides and fertilizers are typically used, or large-scaled livestock farming methods which uses commercial feeds and injects hormones and antibiotics, as unhealthy.

P1: And other foods, I always think fish is a healthy compared to all other types of meats because they are clean, and by clean I mean they are not infested with hormones or injected with antibiotics and things whatever they are, the way they are bred in the sea, it’s full of freedom and life, and one family giving to another family, compared to other ways of farming… where meat is prepared in terms of the domestication of cattle and chicken. Like our Crest Chicken and Rooster Poultry [chicken farms] here. You can see how they are raised in these overcrowded small spaces and even though they say “no hormones, no antibiotics”, how do you think they grow them so big?

P2: And so for that, you know, really, vegetables and fruit is just much better than these canned meat! But like even with vegetables, you need to choose them properly too. You now have the big, big farms that are coming up, that export vegetables too? They use a lot of pesticide and fertilizer. So when marketing I try to buy from the small vendors because I know that they probably don’t use as much, fingers crossed. [Laughs]. And it’s just best to plant your own food.

#### Imported Processed Foods in Comparison Are Unhealthy

Whilst stressing the importance of using locally grown foods and home cooked traditional iTaukei meals, all participants also acknowledged that imported processed foods were unhealthy. However, their discussions on processed foods generally revolved around canned meat and ultra-processed snacks. Two common omissions of processed foods regularly consumed in this sample were noted: (1) sausages, which were often given for school lunch and (2) refined white flour which was a staple in the diet especially for breakfast (as cereals, bread, babakau (Fijian donut) and buns), lunch (typically as sandwiches) and light dinners (also see Section Balancing Healthy and Unhealthy Eating between Meals). It was also noteworthy that only one participant showed preference for wholemeal flour over refined white flour.

P3: I encourage first, my, my children, I’m very strict with how their snacks, like biscuits, potato chips, twisties and stuff. They know it. So, just showing my kids that this is unhealthy and don’t consume this a lot, but have a lot of fruits as well. So my kids love their apples, love their fruits, so I made sure that I buy them and I leave them in the fridge for them. And I pack it with their lunch.

P10: I just tell them, that you have to eat vegetables and things we prepare at home eh. Rather than getting things from somewhere else like the shop, the fish and chips, snacks the… all junk foods. Even the corned mutton… the corned beef. Like that kind of food.

Participants also showed poor understanding of beverages: when discussing healthy beverages, most participants viewed juice made at home using fruit concentrates (e.g., Sunquick and Tang) either in liquid or powder form, as a healthier substitute for carbonated drinks.

P11: We get those Tang sachets or the Sunquick and we have a kumquat tree so at times we make the juice. But that’s not very often. Soft drinks we might have it when we eat out, but not at home. I never buy that because it’s full of sugar. It’s too unhealthy.

### 3.2. How to Eat: Balancing Food Groups and Unhealthy and Healthy Eating

#### 3.2.1. A (Somewhat) Balanced Meal

Another common view of heathy eating offered was that of a balanced diet which has food from three food groups, including carbohydrates, proteins, vitamins and minerals. As participants pointed out, this is what they were taught in school.

P6: You know what’s taught in schools? … To make sure that I have food from all the three food groups in a meal, like a balanced meal … that is what is healthy and nutritious for my family. 

SMB: Can you share what these three food groups are?

P6: Energy giving foods like dalo, cassava, bread…. and um and … body building like fish and eggs and meat… meat, like chicken, beef, pork, mutton. And then there’s health giving foods like our local greens, fruits and vegetables.

However, when participants were further questioned about the quantities in which the three food groups were to be eaten, they admitted being unsure about the quantities and/or shared that they generally did not adhere to the “prescribed” quantities.

SMB: Are there any specific quantities that you need to eat these foods in?

P6: Oh yes, but I don’t think that is followed much. [Laughs]. [] Like when I make soups, and it’s easy to incorporate all three [food groups] in a soup and or a stew. For example, if I make fish soup, I will have fish and local greens and we will have dalo or cassava with it. But to be honest if … if … really look at our plates, we end up eating more of the fish and root crops. Maybe root crops most and fish second and with very little vegetables.

Participants suggested two reasons for this: (1) meat and root crops were generally preferred by family members over vegetables as they were seen as more satiating and tastier, and (2) often meat and at times vegetables (e.g., after natural disasters) were unaffordable and added in lower quantities to their meals. Additionally, as participants described traditional iTaukei foods as healthy, boiled root crops were also viewed as healthy food.

P3: I try to make them eat some vegetables but I know if I put too much in there, they will not eat it. So it has be more potato and sausage and a little bit of carrots, cabbage or a little bit of frozen mixed vegetables to give that color too.

P9: When I cook, I tell them that we are going to be eating a lot of greens… vegetables. Like I make a lot of rourou, with coconut cream. But I tell you, it’s so hard to get them to eat it. Like vegetables. They just dip the boiled cassava in the rourou and eat. They just fill their tummy with the cassava. At least it is boiled so it’s not that unhealthy.

#### 3.2.2. Balancing Unhealthy Eating with Healthy Eating

Mothers acknowledged that their families consumed unhealthy foods and for them healthy eating also meant that they needed to balance unhealthy eating with healthy eating. This balancing of healthy and unhealthy eating happened within meals and between meals.

##### Balancing Healthy and Unhealthy Eating within a Meal

Mothers shared that to counteract what they deemed as unhealthy eating, they would add vegetables and supplement school lunches with fruits to make the meal healthier.

P8: We always try to avoid canned food, canned meats because it’s very fatty. So I try to avoid those. And if I make corned mutton, I make it as a stew so that I can have vegetables in the stew as well, like round cabbage, carrots. So I add something healthy to it. Or if I have corned mutton or beef with cassava, I have boiled local greens with it. So it’s unhealthy but the healthy food makes it less unhealthy. [Laughs].

##### Balancing Healthy and Unhealthy Eating between Meals

Working and studying mothers who often relied on fast food (usually fish and chips or chicken and chips) for lunch shared that they would aim to cook traditional iTaukei cuisine for dinner which they viewed as healthier and gave fruits as snacks to balance out unhealthy eating.

P3: And eating right types of food, like, making sure that not only, like not only during the week you are consuming fast food every day [has fast food for lunch everyday], but also in between you give fruits [as snacks], you have vegetables as well. [] Probably, if I had fast food yesterday, I would have soup today [for dinner].

Mothers also shared that another way in which they balanced unhealthy eating and healthy eating was in relation to portion size and how much was eaten. Mothers shared that they served lighter dinner to compensate for having eaten too much for breakfast and/or lunch. In this sample, mothers would serve “lighter dinners” a minimum of once a week and up to five nights a week. However, mothers who opted to make “lighter dinners” for their families often made dinners that consisted of foods high in carbohydrates, such as bread, babakau (Fijian donuts), pies, root crops (cassava and dalo), usually served with tea.

P9: To me healthy eating is like mostly,… having a healthy breakfast, a really good breakfast and a light lunch and sort of like a not that heavy dinner. Like just tea and snacks for dinner. Like we can have babakau or sandwiches or leftover root crops with tea for dinner.

### 3.3. Reasons for Healthy Eating

Healthy eating was also viewed by mothers as meals and meal patterns that support health, wellbeing and meet lifestyle requirements. Mothers who experienced illness such as cancer (P4), or more commonly, had family members living with them who were ill or were suffering from chronic illness, such as high blood pressure or diabetes, described healthy eating as eating foods that help with the management of these pre-existing health conditions. Mothers also described healthy eating as a way of protecting against diseases. Two of the mothers even suggested that the amount of food eaten was another way of viewing healthy eating as overeating can result in unhealthy weight gain which, in turn, can have negative consequences on health.

P4: When I was diagnosed with cancer, there’s a few things that I had to let go of which I feel contributes more into the disease itself, so I have let go of sugar, dairy foods and I am actually gluten and dairy intolerant. So right now my body needs more greens, a lot of iron foods like liver, red meat and fish. So with that my whole family has decided to eat similar meals because it’s healthier and also as a support for my health and wellbeing.

P12: Like to be honest this trying to include vegetables in our diet comes from my mother because she had a health scare recently, she is a diabetic, and so we are looking at our diets and what we can do to improve it. My mum is a heavy eater and really indulges in a lot of oily foods and so while we weren’t eating vegetables before we are trying to incorporate it more in our diet now. We are more health conscious since she became sick. We also control how much we eat because she has reduce weight.

Mothers also shared that healthy eating would support the desired lifestyle of family members, such as for work, studies, sports and weight management. To this end, some mothers stressed the importance of a “wholesome breakfast” or a “heavy breakfast” which they associated with giving family members enough energy for the day. 

P1: Eating a wholesome breakfast is very important for us. And realize, when I eat good breakfast, I perform well at work. When I don’t eat a good breakfast, I’m not really performing that well, I’m underperforming. [Laughs].

P5: Also, I don’t give them money to buy junks [ultra-processed snacks] from the canteen and I pack a good lunch, two lunch boxes for them and give them fruits for snacks [apples and pears] so they can have that in recess. Because they need to eat well, eat healthy to study well. If they eat too much sugar during recess, they will feel sleepy and can’t study.

## 4. Discussion

This study expanded our understanding of the HFE of urban indigenous Fijians by exploring how iTaukei mothers perceive healthy eating and how these perceptions impact the food decisions they make for their families. Similar to previous studies, it was found that the participants of this study held complex, multifaceted views of healthy eating, e.g., [35,39,40]. Furthermore, these perceptions impacted (1) their food choice, including, the types of foods that should be eaten and avoided, (2) the strategies that were adopted for ensuring healthy eating in their households and (3) the perceived motivators of healthy eating. This study also confirmed the views on healthy eating noted in other studies where healthy eating was not only viewed as foods that should be eaten but also encompassed considerations around food quality, preparation methods, nutrient composition, concepts of balance and eating foods that promote health, well-being and support lifestyle needs [33,34,35,36,37,38,39,40]. However, in addition to the aforementioned perceptions of healthy eating, the participants in this study distinctively placed much importance on traditional (iTaukei) cuisine and farming methods. This finding also indicates iTaukei mothers’ awareness of nutrition transitions in their communities from traditional plant-based diets to diets high in imported processed foods. It also demonstrates the active steps iTaukei mothers are taking to minimize the impacts of nutrition transitions on their HFE through inclusion of traditional cuisine and emphasizing locally farmed foods.

Consistent with previous studies, e.g., [26], the findings of this study also indicated that mothers’ perceptions on healthy eating impacted both positive and negative family food decisions, and ultimately their HFE. On one hand, mothers’ views on healthy eating meant that they undertook concerted efforts to prepare healthy meals and set healthy eating practices within their families by including fruits and vegetables into diets and avoiding canned meat, ultra-processed snacks and carbonated drinks. On the other hand, inaccurate beliefs or limitations in nutritional knowledge led to unhealthy eating practices such as poorly controlled consumption of refined white flour, boiled root crops, substituting fruit concentrates for carbonated drinks and serving lighter dinners consisting mainly of carbohydrates.

The findings of this study also indicated that perceptions of healthy eating impacted the strategies that the mothers used to promote healthy eating within their families. Similar to studies reviewed by Paquette [40], mothers in this study also shared how they attempted to balance out unhealthy eating with healthy eating by adding more fruits and vegetables in the diet, balance eating out with eating homemade traditional iTaukei cuisine and counteract overeating during breakfast or lunch by serving light dinners. Finally, consistent with the previous literature, this study also recorded the crucial role that iTaukei mothers play as sociocultural agents who set eating-appropriateness standards for their families, e.g., [9,19,20,21,22,23,24], and mediate the impacts of environmental factors such as accessibility and availability through the food choices they make. The latter was especially evident when mothers opted for what they perceived as healthier food options, even though unhealthy options were available.

### 4.1. Implications and Opportunities for Public Health Interventions

The findings of this study highlight several opportunities for nutrition and public health interventions and provide an insight on the impact of current interventions on eating within this group.

#### 4.1.1. Capitalizing on the Emphasis on Traditional Cuisine and Farming

A prominent finding of this study was that the participants closely associated healthy eating and healthy foods with traditional iTaukei cuisine and traditional ways of producing foods. Traditional iTaukei cuisine was described as soups and stews containing meat or fish and locally grown vegetables with boiled root crops as sides. As participants pointed out, such foods are low in oil and easily incorporate foods from all three food groups. The alarmingly high rates of childhood and adult obesity in Pacifica communities and the consequent increase in non-communicable diseases has been, in part, attributed to nutrition transitions from traditional plant-based diets to diets high in ultra-processed foods [9,10,11]. This finding signals value systems idealizing traditional foods and, therefore, the barriers to the uptake of traditional foods need to be further explored. The findings of this study indicate that one potential barrier to consumption of traditional iTaukei cuisine in this sample might be that since traditional iTaukei food is generally cooked in liquids, they are not suitable for school or work lunches. Presumably this was the reason that most mothers in this sample made a variety of sandwiches, sausages, boiled root crops and noodles for school lunches and working parents or mothers resorted to fast foods for lunch. This finding suggests that investment in food education and training for mothers in providing healthier alternatives may improve HFE in this community [26]. Furthermore, the emphasis that participants placed on local produce and farming systems suggests opportunities for investment in home food procurement skills to increase dietary diversity and food security within this community [19]. At the national level, strengthening and increasing the visibility of health campaigns, e.g., [49], that encourage consumption of locally grown foods, can improve Fijian diets and combat nutrition transition in Fiji.

#### 4.1.2. Addressing Poor Understanding of Food Composition, Processed Foods and Healthy Eating Campaigns

When discussing their perceptions of healthy eating, serious omissions and inaccurate assumptions were apparent which led to unhealthy food decisions and negatively impacted the HFE. Participants not only showed little compliance in eating the three food groups in the appropriate proportions, but also shared that they lacked understanding of what these portions should be. This limited understanding showed in their typical meals which prominently featured carbohydrates and little of other food groups. Participants also shared that they relied heavily on carbohydrate dense foods, which can be one of the contributors to the high rates of obesity and overweight observed in this community [8,11]. Furthermore, participants’ characterization of processed foods was limited to canned meat, ultra-processed snacks and carbonated drinks. While these findings indicate that healthy eating campaigns such as importance of a balanced diet and avoiding carbonated drinks, canned meat and ultra-processed snacks appears to be working within this sample, it also meant that the more common processed foods consumed in this sample such as refined white flour were being overlooked. This was particularly concerning since refined flour and sugar are major components of breakfasts, lunch and “lighter dinners”. Increasing nutrition literacy and targeting these misconceptions has potential to result in healthier dietary choices within this group [26].

Healthy eating campaigns that emphasize eating a good breakfast and having a light dinner also seems to have been taken on board by the participants of this study and mothers emphasized the importance of wholesome breakfasts. However, the notion of “lighter dinners” has been translated into serving carbohydrate dense foods that don’t require much cooking, (e.g., white bread, babakau (Fijian donuts), pies and boiled root crops). In an earlier study, we noted that this practice of substituting dinners with carbohydrate dense foods to compensate for overeating increased during COVID-19 lockdowns when people were confined to their homes [19] and we expect that a similar pattern might exist during holidays. Therefore, this community might greatly benefit from targeted interventions to address this misinterpretation of “lighter dinners”. 

Fijian schools also encourage inclusion of fruits as snacks for school children [50], and participants in this sample often spoke about how they packed fruits like apples and pears as snacks to encourage healthy eating and to balance out unhealthy eating. However, it was noted that this emphasis on fruit consumption was restricted to children and was absent from the diets of adults of most families. It was also noticed that working parents and children often ate differently for lunch, with a higher consumption of fast food amongst working parents than their children. This finding suggests that while school-based healthy eating campaigns appear to be working in this sample, health messaging targeting fruit and vegetable consumption for adults and reduction in fast food in this community, needs to be evaluated.

These findings highlight the need for targeted efforts to improve nutritional knowledge, promote balanced meals and address misconceptions around the messaging of healthy eating campaigns implemented by the Fijian government. Importantly, food-based dietary guidelines developed by Fiji’s National Food and Nutrition Center [49] need to be updated on the basis of local research and made more accessible to the Fijian community.

### 4.2. Strengths and Limitations

This study was the first of its kind to undertake an in-depth exploration of the views of Pacifica mothers on healthy eating and provides an insight on how these views impact the food decisions they perform for their families. The study nonetheless has several limitations that need to be considered in interpreting its findings. First, the findings of this study cannot be generalized beyond this sample and the method of data collection used is open to social-desirability and other biases associated with self-reports. Given the prominence of the findings, more research is required to triangulate the findings with larger samples in relation to how perceptions on healthy eating impact food choice and the HFE within this community. Second, while the study considered perceptions of healthy eating and how it impacted family food decisions, it is clear that participants made meal decisions based on other factors such as food costs, preferences and convenience, which need to be explored further.

## 5. Conclusions

Mothers are important sociocultural agents who play critical roles in their HFE through setting eating-appropriateness standards and mitigating impacts of food availability and accessibility in the HFE. Mothers in this study held complex, multifaceted perceptions on healthy eating and these perceptions had both positive and negative impacts on the family food choices they made, the strategies they adopted for healthy eating and their perceived motivators for healthy eating. Investment in nutrition literacy that targets misconceptions about healthy eating, and food education and training in order to upskill mothers to provide healthier alternatives, may improve the HFE in this community. The findings of this study also underscore the need for a deeper understanding and analysis of the uptake of public health messaging related to healthy and unhealthy eating, and the importance of targeted promotion of healthful nutrition in this community and more broadly in the Pacific. Furthermore, promoting consumption of traditional and locally grown foods can enhance nutrition and food security and combat nutrition transition in the region. 

## Data Availability

The data presented in this study are not publicly available due to restrictions of the informed consent obtained for this study.
